# Apoptosis of viral-infected airway epithelial cells limit viral production and is altered by corticosteroid exposure

**DOI:** 10.1186/1465-9921-7-78

**Published:** 2006-05-18

**Authors:** Gurpreet K Singhera, Tiffany S Chan, Jenny Y Cheng, Timothy Z Vitalis, Kimm J Hamann, Delbert R Dorscheid

**Affiliations:** 1 The James Hogg iCAPTURE Centre for Cardiovascular and Pulmonary Research/ Critical Care Group, St. Paul's Hospital, University of British Columbia, Vancouver, British Columbia, V6Z-1Y6, Canada; 2 Michael Smith Laboratories, 2185 East Mall, Vancouver, BC, V6T 1Z4, Canada; 3 Section of Pulmonary and Critical Care Medicine, University of Chicago, Chicago, IL, Zip Code 60637, USA

## Abstract

**Background:**

Effects of respiratory viral infection on airway epithelium include airway hyper-responsiveness and inflammation. Both features may contribute to the development of asthma. Excessive damage and loss of epithelial cells are characteristic in asthma and may result from viral infection.

**Objective:**

To investigate apoptosis in Adenoviral-infected Guinea pigs and determine the role of death receptor and ligand expression in the airway epithelial response to limit viral infection.

**Methods:**

Animal models included both an Acute and a Chronic Adeno-infection with ovalbumin-induced airway inflammation with/without corticosteroid treatment. Isolated airway epithelial cells were cultured to study viral production after infection under similar conditions. Immunohistochemistry, western blots and viral DNA detection were used to assess apoptosis, death receptor and TRAIL expression and viral release.

**Results:**

*In vivo *and *in vitro *Adeno-infection demonstrated different apoptotic and death receptors (DR) 4 and 5 expression in response to corticosteroid exposure. In the Acute Adeno-infection model, apoptosis and DR4/5 expression was coordinated and were time-dependent. However, *in vitro *Acute viral infection in the presence of corticosteroids demonstrated delayed apoptosis and prolonged viral particle production. This reduction in apoptosis in Adeno-infected epithelial cells by corticosteroids exposure induced a prolonged virus production via both DR4 and TRAIL protein suppression. In the Chronic model where animals were ovalbumin-sensitized/challenged and were treated with corticosteroids, apoptosis was reduced relative to adenovirus-infected or corticosteroid alone.

**Conclusion:**

Our data suggests that apoptosis of infected cells limits viral production and may be mediated by DR4/5 and TRAIL expression. In the Acute model of Adeno-infection, corticosteroid exposure may prolong viral particle production by altering this apoptotic response of the infected cells. This results from decreased DR4 and TRAIL expression. In the Chronic model treated with corticosteroids, a similar decreased apoptosis was observed. This data suggests that DR and TRAIL modulation by corticosteroids may be important in viral infection of airway epithelium. The prolonged virus release in the setting of corticosteroids may result from reduced apoptosis and suppressed DR4/TRAIL expression by the infected cells.

## Background

Viral respiratory tract infections have been implicated in several ways with the pathogenesis of asthma. These include the initial onset of asthma, particularly in the context of post-bronchiolitis wheezing and asthma after hospitalization for respiratory syncytcial virus (RSV) [1] and in asthma chronicity and steroid resistance in Ad5 infections [2]. Ad5 infections are epidemiologically important, and are estimated to cause ~5–10% of childhood respiratory infections [3]. Despite well-established epidemiological associations between infections by viruses and the development of asthma, the mechanisms by which these pathogens contribute to the etiology of asthma are poorly understood.

Apoptosis(programmed cell death) is a common cellular response to virus infection [4]. Cell culture studies have established that many common respiratory viruses can induce apoptosis in epithelial cells [5,6]. Recent work has demonstrated that viral infections can activate the tumour necrosis factor (TNF)-related apoptosis-inducing ligand (TRAIL) pathway, which leads to the selective apoptosis of virus-infected cells [7]. TRAIL is the ligand for members of the TNF-α death receptor (DR) family that includes molecules such as DR4 and DR5 [8]. Presently there are limited data available about the expression of TRAIL and DR in normal or viral infected airway tissues. These studies were undertaken to examine the role of Ad5 infection on expression and function of TRAIL receptors DR4 and DR5.

The first objective of this study was to determine the baseline and viral-induced expression of DR4/DR5 in Ad5 infected Guinea pig lungs and to correlate this expression to apoptosis of the infected airway epithelial cells (AEC). In some situations apoptosis can contribute to pathogenesis, but more typically it is an important factor in the host defence mechanism which hastens the death of infected cells to limit the replication and spread of virus [8]. In healthy tissues, apoptosis is highly regulated to maintain tissue integrity, function, and turnover of cells; therefore it is generally viewed as being an anti-inflammatory process. The role for apoptosis in the setting of viral infections consequently may be a mechanism to limit the extent of infection, including inflammation.

Our next objective was to determine whether DR4/DR5 expression and apoptosis of infected epithelial cells has a role in viral infections by Guinea pig airway epithelium and how this may be altered by corticosteroid exposure. This objective was based on reports regarding the rate of viral detection as higher in asthmatic children than non-asthmatics, symptomatic or not, suggesting a possible susceptibility to longer viral infections particularly in cases of steroid resistance [9-11]. The present study was designed to determine the role of apoptosis and DR expression in models of airway inflammation and viral infection of airway epithelial cells. Our data suggest a role for DR in limiting Acute virus infection through apoptosis of infected cells. Modulation of DR and its ligand TRAIL expression is effected by corticosteroids exposure and may be implicated as a potential mechanism of viral persistence in the airway epithelium. Steroid treatment prolonged virus release from airway epithelial cells coordinate with the reduced DR4 and TRAIL expression and altered the apoptosis of infected airway cells. Dysregulation of this apoptotic process may contribute to airway remodeling.

## Methods

### Animals

Female Guinea pigs *Cavia porcellus *(Cam Hartley strain), weighing 250–300 g (Charles River, ON, Canada) were housed in polycarbonate cages fitted with high efficiency particulate air filter covers. The animals were provided care as approved by the University of British Columbia Animal Care Committee, following published guidelines of the Canadian Council on Animal Care.

### Adenoviral infection

#### Acute model

**Figure 1:**
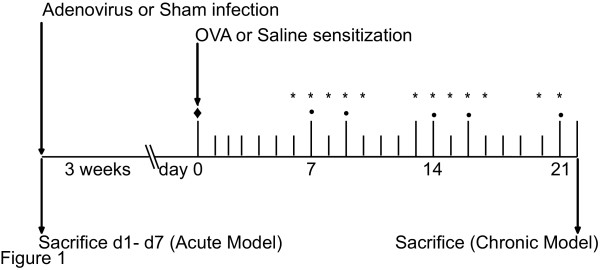
**Study design for Ad5 infection and allergic inflammation in Cam Hartley Guinea pigs **Animal model as modified from [12]. Animals were Ad5 infected or sham treated. In Acute model animals were sacrificed at d1–d7 post-infection. Other Ad5 infected or Sham treated animals were supported for 3 weeks post-infection (Chronic model). These Guinea pigs were then sensitized with OVA by aerosol administration at day 0 (◆) followed by aerosol challenges as indicated (*). Steroids were given to a subset of these Guinea pigs at the indicated days (•) to permit resolution of the OVA-induced inflammation

Guinea pigs were anesthetized with 4% halothane balanced with oxygen and were either adeno virus (Ad5) infected via intranasal instillation or sham treated as previously described [12]. For the Acute model (Figure 1), animals were sacrificed at 1, 3, 4 and 7 days post-infection (dPi).

#### Chronic model: allergen-induced lung inflammation and Ad5 infection

For the Chronic model (Figure 1), three weeks after Ad5 infection, half of Ad5-infected and Sham-treated animals were sensitized with ovalbumin (OVA) by exposure for 10 minutes to an aerosol spray of 1% OVA with 4% (vol/vol) heat-killed pertussis vaccine in normal saline solution followed by challenge consisted of delivering an aerosol spray of 0.5% OVA (wt/vol) solution over a 5-minute period. The remaining Sham animals were sensitized to normal saline containing 4% heat-killed pertussis vaccine and served as control animals for allergen sensitization and challenged with normal saline solution. Diphenhydramine (0.2 ml of 40 mg/ml in normal saline solution) was administered intraperitoneally 1 h before each OVA challenge to prevent anaphylactic shock. One group of Ad5 infected/OVA sensitized/challenged animals were injected with Budesonide (Bud) (20 mg/kg) intra-peritoneal on 12 occasions over 16 days starting 24 hours before the first allergen challenge. Three hours after the last OVA challenge or saline exposure, Guinea pigs were sacrificed with sodium pentobarbitol administered intra-peritoneally. Lungs from the each treatment group were then processed. Final groups included Sham control, Ad5, Bud, OVA, OVA+ Bud, OVA+ Bud+ Ad5 groups in the Chronic model.

### Tissue preparation and immunohistochemistry

The right lung was separated from the main stem bronchus, weighed and then inflated with 50% Optimal Cutting Temperature compound (Tissue Tek, Miles Inc) in PBS (pH 7.4). The inflated right lung lobe was cut into 3 blocks in the transverse plane, fixed in buffered 10% formalin and processed into paraffin. Immunohistochemical (IHC) studies of paraffin-embedded formalin-fixed tissue sections followed standard protocol of antigen retrieval with autoclaving in 1X Citra buffer (BioGenex, CA) or Trypsin digestion and blocking with Universal blocking solution from DAKO (ON, Canada). Polyclonal rabbit anti-DR4 and -DR5 antibodies (Cell Sciences Inc, MA) and rabbit anti- PARP p85 fragment antibody (Promega, MA) were used along with normal Rabbit IgG as negative control to measure receptor expression and apoptosis respectively. p85-PARP antibody is specific for the p85 fragment of PARP generated by caspase cleavage and provides a reliable measure of *in situ *apoptosis [13]. Antibody binding was detected using avidin-biotin complex method with naphthol AS-BI and New fuchsin as substrate as per DAKO cytomation protocol. A semi-quantitative scoring method was used by three independent blinded observers to record DR4 and DR5 staining intensity by scoring from scale of 0–4 (0- being no staining, and 4- being maximum staining) depending on staining intensity in circular, medium sized airways. For p85-PARP, the total number of positive cells in a minimum of 3 airways, scored by three independent observers, were determined and scored as a percentage. The mean score from 4 sections for each treatment was used to assign the final score for the staining of all antibodies on all the sections.

### Immunohistochemistry for E1A staining of Adeno-infected guinea pig lung tissue

Immunohistochemical studies of formalin-fixed paraffin embedded tissue sections followed standard protocol of antigen retrieval with autoclaving in 6M Urea and blocking with Universal blocking solution from DAKO (ON, Canada). Anti-adenovirus E1A mouse monoclonal antibody (Calbiochem) was used along with normal mouse IgG as negative control to detect E1A protein. Antibody binding was detected using APPAP method (DAKO) with naphthol AS-BI and New fuchsin as substrate as per DAKO cytomation protocol without any counterstaining.

### Guinea Pig Tracheal Epithelial Cell (GPTEC) isolation

Mid-cervical tracheas were dissected under sterile conditions, and placed into 0.1% protease solution (type 25 from *Bacillus polymyxia *Sigma-Aldrich ON, Canada) in HBSS for two hrs at 37°C[14]. Tracheal segments were then transferred to plates containing Ham's F12 medium (Sigma-Aldrich, ON) with 5% FCS. Epithelial cells were dislodged using a micro-spatula, triturated through a small-bore pipette tip and centrifuged at 850 × g for 11 min, then washed twice and GPTEC were maintained and epithelial cell origin was confirmed as per protocol [15]. At 90–100% confluency cultured GPTEC were infected with Ad5 at the multiplicity of infection of 10 (MOI _10_). Uninfected GPTEC served as a Sham control. Conditioned media was collected daily to analyze the released viral particle production and fresh media was added.

#### Western blots

Western blots were done as previously described [16]. Membranes were probed for DR4, TRAIL and PARP proteins using polyclonal anti-DR4 antibody (BD Pharmingen), polyclonal anti-TRAIL antibody (eBiosciences) and monoclonal anti -PARP antibody (BioMol Research labs) respectively. Membranes were reprobed with an antibody for β-actin (Sigma) when appropriate to control for equal protein loading. Densitometry was performed to quantitate expression.

### Picogreen assay for nucleic acid quantitaion to determine viral particle number

The amount of Ad5 released into the conditioned media was determined by the quantity of detected viral DNA [17] using Picogreen (Invitrogen Canada, ON) as per kit instructions. The viral DNA concentration was converted to viral particles/ml (VP/ml) using the equation: VP/ml= DNA conc. (ng/ml) X (2.6 × 10^8 ^VP/ml/10.3 ng/ml).

### Adenoviral PCR

PCR was performed on the conditioned media collected from the Ad5 infected GPTEC to confirm the detected DNA was viral in origin. Primers were specific for Ad5 virus [F-primer: 5' – GCCGCGTGGTTTACATGCACATC 3' and R-primer: 5' – CAGCACGCCGCGGATGTCAAA GT3'] [18].

### Statistical analysis

Values are presented as means ± SE. The significance of differences between means was assessed by Mann-Whitney test with the level of significance set at p ≤ 0.05 to compare the unpaired populations where sample size is small and therefore Gaussian distribution cannot be assumed. All statistical analyses were performed using Prism 3 software.

## Results

### Guinea pig model of ovalbumin (OVA)-induced inflammation and corticosteroid treatment in 6-weeks Ad5 infected Guinea pigs

Animals were created per model described in the Methods and used by others [12]. The model of OVA-induced inflammation generates changes in the airway compatible to inflammatory diseases such as asthma.

**Figure 2:**
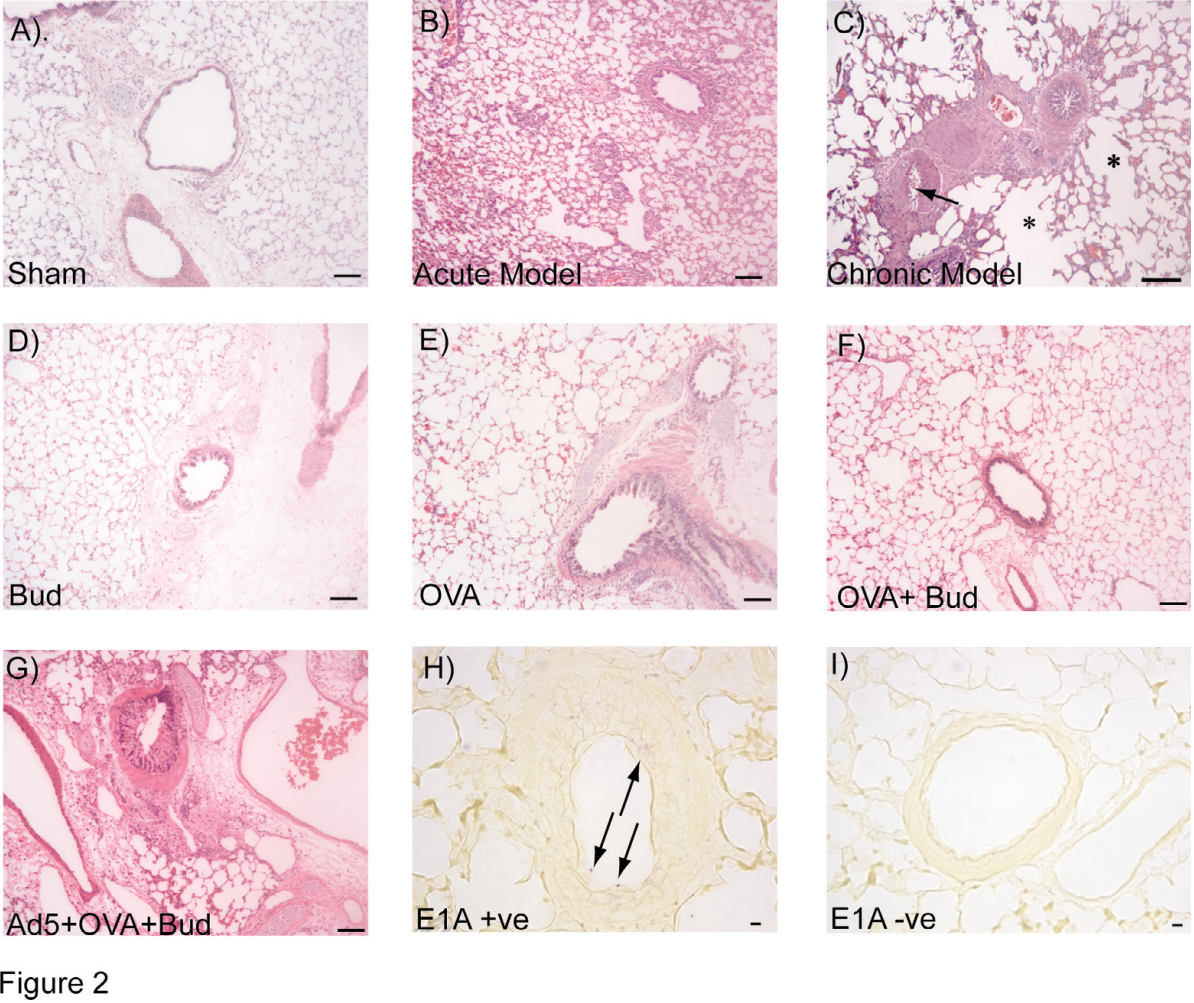
**Hematoxylin and eosin-stained representative lung sections from the Guinea pig models**. Guinea pig lungs sections from sham control (A); lung section after 7 days post-Ad5 infection (Acute model) (B); and after 6 weeks post-Ad5 infection (Chronic model) (C). Arrow indicate regions of inflammation and eosinophilic infiltration, also noted in the Chronic model is the damage to the alveolar parenchyma as indicated by *. Guinea pig airways showing normal histology in Budesonide (Bud) treated lungs (D), whereas ovalbumin (OVA) sensitized/challenged lung sections show airway inflammation and smooth muscle hypertrophy (E). OVA+Bud treated lung section had little eosinophilic infilteration, and no hypertrophy (F). The Ad5+OVA+Bud treated lungs (G) demonstrate damage in the alveolar space, inflammation and alterations of other airway wall components. E1A protein was detected in chronically infected lung sections, arrows indicate positive staining for E1A protein in the nuclei of airway epithelial cells (H) when compared to no staining for isotype control (I). Scale bar represent 100 μm in panels A through G, and 10 μm for panel H and panel I.

Hematoxylin and eosin (H&E) stained Guinea pig lung tissue sections demonstrated histological changes coordinate with the various treatment groups (Figure 2). Sham treated control lung sections demonstrated normal histology (panel A). All shams (either single or in combination) demonstrated no histological changes. Similarly there was no change in DR expression; hence only one ''representative'' sham is shown. Adeno-viral infection generated an eosinophilic infiltration and inflammation in the Acute model (panel B). In the Chronic model of infection damage in the alveolar parenchyma was noted along with more extensive inflammation (panel C). Bud treatment yielded Guinea pig lung sections with near normal histology and no significant inflammation (panel D), whereas inflammation and smooth muscle hypertrophy was observed in the OVA-sensitized/challenged lung sections (panel E). OVA+Bud treated Guinea pig lung section had little eosinophilic infiltration, and no smooth muscle hypertrophy compared to OVA alone group (panel F). The combination of Ad5+OVA+Bud in the Guinea pig lung demonstrated damage in the alveolar parenchyma, inflammation and smooth muscle hypertrophy (panel G). Viral persistence in terms of E1A protein expression was detected in the Chronic model of Guinea pig airway epithelial cells as indicated by pink staining of the nuclei (panel H) compared to no stain for the isotype control (panel I). Arrows indicated positive staining for E1A protein in the airways.

### Apoptosis and DR4/DR5 expression in acute model of adenoviral infection

**Figure 3:**
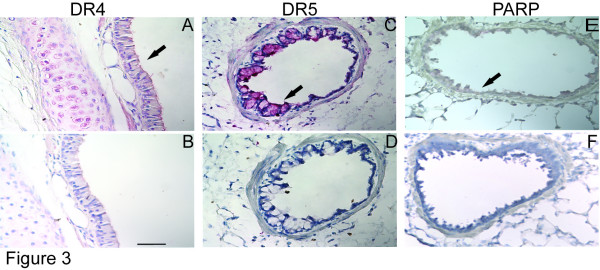
**Representative images of airway epithelial immunostaining of Cam Hartley Guinea pigs **Semi-quantitative scoring was utilized to determine the expression for DR4(A), DR5 (C) and p85-PARP (E) in immunohistochemically stained lung sections. Panels B, D and F were the isotype controls for the respective antibodies. Arrows indicate the stained epithelial cells. Scale bar represent 10 μm in panels A through F.

**Figure 4:**
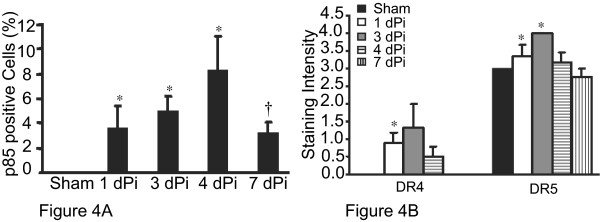
**Acutely infected GPTEC demonstrate apoptosis coordinate with DR4 and DR5 expression **Semi-quantitative scoring was utilized to determine the expression of p85-PARP, DR4 and DR5 in the Guinea pig lung sections by immunohistochemistry. p85-PARP was significantly higher in 1 -4 dPi lung sections compared to Sham controls, peaked at 4 dPi and decreasing significantly by 7 dPi (Figure 4A). This trend in apoptosis in the Acute model was coordinate with the changes in DR4 and DR5 expression (Figure 4B). * p < 0.05 compared to Sham and †p < 0.05 compared to 4 dPi.

Both DR4 and DR5 were expressed in the airway epithelium of Guinea pigs after viral infection and OVA sensitization and challenge as demonstrated by representative images of DR4, DR5 and p85-PARP immunohistochemical staining (Figure 3). Apoptosis and death receptor expression were observed as a result of Acute Ad5 infection of the airway epithelium. For this model lung tissues were collected up to 7 days after the initial Ad5 infection and assessed for p85-PARP staining as a marker of apoptosis. Positive staining for the p85 fragment (Figure 4A) increased from 1 day post-infection (dPi) (3.7% ± 2.4%) to 4 dPi (8.3% ± 2.6%) and reduced by 7 dPi (3.2% ± 1.1%). There was a significant increase (* p < 0.05) in apoptosis for 1 dPi, 3 dPi and 4 dPi samples compared to uninfected Sham control. After its peak expression at 4 dPi, apoptosis decreased significantly by 7 dPi († p < 0.05) compared to 4 dPi and this 7 dPi apoptosis was not significantly different from Sham (Figure 4A).

Both DR4 and DR5 were detected in the Acute infection. No DR4 expression was detected at baseline in the Sham controls. However, after Acute Ad5 infection DR4 expression peaked at 3 dPi (1.3 ± 0.7) and returned towards baseline at 7 dPi (Figure 4B). DR5 was expressed in the Sham control and from 1–7 dPi (Figure 4B). This increased expression is noted after Ad5 infection, and maximal expression occurred at 3 dPi. The apoptosis observed in the Acute model was in concordance with the DR4/DR5 expression (Figure 4A/4B). The apoptotic response lags behind the increased DR4/DR5 expression, as might be expected. When the DR4/5 expression was correlated with apoptosis at the succeeding time point, there was a significant positive correlation (p = 0.01 for DR4 vs. apoptosis and p = 0.0001 for DR5 vs. apoptosis). This data suggests that apoptosis in Acute vial infection may be mediated by DR4/DR5 signaling.

### *In vitro *apoptosis and death receptor expression after acute Ad5 virus infection

**Figure 5:**
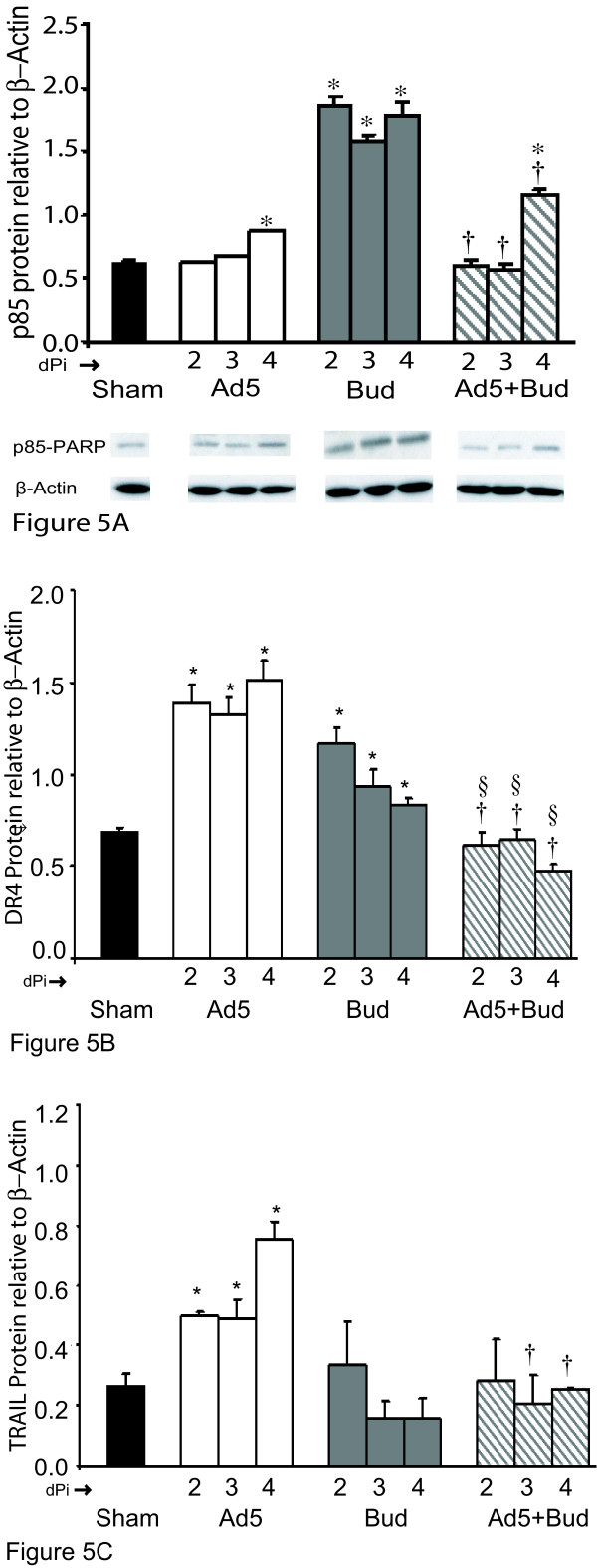
**in vitro model of Ad5-infected GPTEC demonstrate p85-PARP, DR4 and TRAIL expression**. Western blotting of total protein lysates collected from Ad5 infected GPTEC demonstrated elevated apoptosis in Ad5 infected cells by 4 dPi and Bud treated cells from 2- 4 dPi compared to Sham cells. Ad5+ Bud group had significantly less apoptosis compared to Bud alone. The inset shows protein bands corresponding to p85-PARP and house keeping β-Actin protein for respective groups (Figure 5A). Ad5 induced apoptosis corresponds to DR4 expression (Figure 5B) and to DR ligand TRAIL (Figure 5C). Ad5+Bud group demonstrated suppressed DR4 and TRAIL protein expression compared to Ad5 alone and Bud alone for respective treatment days (Figure 5B, 5C), * p < 0.05 compared to Sham, §p < 0.05 compared to Bud alone, † p < 0.05 compared to Ad5 alone.

From the Acute animal studies we observed that apoptosis of the viral infected cells may be a mechanism to limit the infection. DR4 and DR5 were noted to be expressed and regulated in response to the Acute viral infection. To validate this model we established a cell culture system using GPTEC isolated from the Cam Hartley Guinea pigs. GPTEC were infected with Ad5 virus at MOI_10 _to effect maximal infection of ~20% of the cultured cells. At 1 dPi (Ad5 or Sham) the GPTEC were treated with +/- Bud to examine the effect of corticosteroids. Untreated cultured cells served as Sham controls. As determined by detection of p85-PARP protein expression, apoptosis increased after Ad5 infection when compared to uninfected Sham controls (Figure 5A). The detection of the p85 fragment at 4 dPi was higher than the uninfected cells (0.9 ± 0.01 vs. 0.6 ± 0.02; * p ≤ 0.05). Bud-treated GPTEC had the highest p85-PARP detection (1.8 ± 0.07 Bud 2d, 1.6 ± 0.05 Bud 3d, and 1.8 ± 0.1 Bud 4d) and all time points were significantly increased from Sham (* p ≤ 0.05). This is consistent with corticosteroid-induced apoptosis of AEC as demonstrated previously [16] and is independent from death receptor expression and function. The Ad5+Bud group demonstrated a reduction in apoptosis († p ≤ 0.05) when compared to Bud alone. The early trend of apoptosis in Acute Ad5 infection was absent in the Ad5+Bud group, although by day 4 (4 dPi) apoptosis was significantly increased (1.2 + 0.03 * p ≤ 0.05) compared to Sham (Figure 5A). Overall with a low infection rate the absolute changes in detected apoptosis may remain low; however any change in the timing of apoptosis could have significant effects later. To observe the affect of Bud exposure on the Ad5 infection and related apoptosis, p85 affect for both Ad5 and Ad5+Bud groups was normalized to the baseline (Ad5 1 dPi). Table 1 demonstrates that Ad5 infection demonstrated an "early" initiation of apoptosis: 11.3% increase at 2 dPi, 18.2% at 3 dPi and 57% by 4 dPi, whereas Ad5+Bud demonstrated a "late" initiation of apoptosis effect starting at 7% at 2 dPi, only 1.6% at 3 dPi and 107% at 4 dPi. Overall apoptosis is similar between Ad5 and Ad5+Bud; however the trend for increasing apoptosis over 4 days after Ad5 infection was significantly altered. This alteration by Bud in the pattern of apoptosis of Ad5 infected GPTEC is in association with Bud suppressing DR4 and TRAIL expression when compared to Ad5 alone (Figure 5B, 5C).

Our Guinea pig Acute infection model, suggested a role for both DR4 and DR5 in the modulation of apoptosis. We focused on DR4 as a major candidate in our *in vitro *Ad5-infection model as the magnitude of change in DR4 expression was much higher than that of DR5. There was baseline DR4 expression in the uninfected Sham controls that was significantly less than the Ad5 infected cells for all time points. Figure 5B demonstrates that DR4 protein was significantly increased (* p < 0.05) in Ad5 alone and Bud alone groups from Sham baseline at 2–4 dPi. The Ad5 infected group demonstrated the highest DR4 expression at 4 dPi (1.5 ± 0.1). DR4 expression was altered in the presence of corticosteroids. Bud (1 μM) was added to the cultured GPTEC at 1 dPi to model the treatment for the resolution of the virus-induced inflammation. As demonstrated in Figure 5B there is an initial increase in DR4 expression after 2d of Bud exposure (1.16 ± 0.09 vs. 0.68 + 0.02 p < 0.05) however the magnitude of increase in DR4 expression did not persist over time (3d 0.93 ± 0.09 vs. Sham * p ≤ 0.05; 4d 0.83 ± 0.03 vs. Sham p < 0.05), and at all time points expression was greater than Sham (* p < 0.05). Ad5+Bud demonstrated a significant reduction in DR4 expression when compared to either Ad5 alone († p < 0.05) or Bud alone (§p < 0.05). The DR4 expression in Ad5+Bud was not different from the Sham control. The individual challenges of either Ad5 or Bud increased DR4 expression within 1d; however the combination was not synergistic.

TRAIL is the ligand for the receptors DR4 and DR5. TRAIL protein expression was determined in the total protein lysates obtained from the Ad5, Bud and Ad5+Bud treated GPTEC. Ad5 alone treated cells demonstrated the significant increase in TRAIL expression (Figure 5C) at 2–3 dPi increasing further at 4 dPi (0.75 ± 0.05 * p ≤ 0.05) which mirrors the effect on DR4 expression. TRAIL expression was not increased by Bud alone and Bud+Ad5 treatments demonstrated a significant reduction in TRAIL expression at day 3 and day 4 when compared to Ad5 alone († p ≤ 0.05). This alteration by Bud in the pattern of apoptosis of Ad5 infected GPTEC is in association with Bud suppressing both DR4 and TRAIL expression when compared to Ad5 alone.

**Table 1:** 

**Groups**	**Day 2**	**Day 3**	**Day 4**
Ad5	11.3	18.2	56.9
Ad + Bud	6.9	1.6	107

### Viral particle release by Ad5 infected GPTEC *in vitro*

**Figure 6:**
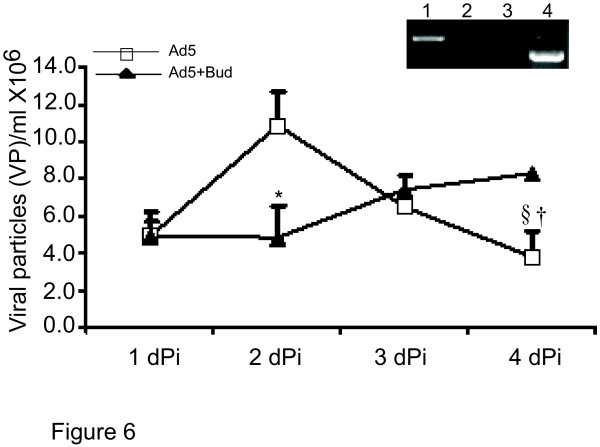
**Prolonged viral particle release into culture media is coordinate with exposure to corticosteroids **Picogreen assay performed on the condition media collected from Ad5 infected GPTEC with/out Bud exposure show an early reduction in viral particle release as determined by the detection of Ad5 DNA in the conditioned media. However beyond this initial 24 h period of treatment the detection of viral DNA continued to increase while in the untreated Ad5 infected GPTEC the detection of viral DNA significantly reduced. The insert demonstrates amplification of WtAd5 gene (Lane 1), and housekeeping β-actin (Lane 2) from the conditioned media, and WtAd5 gene (Lane 3), and human β-actin (Lane 4) from the Sham infected human airway epithelial cells. This demonstrates that amplified signal in Lane 1 is specific from the viral DNA and not from the epithelial cells in the supernatant. * p < 0.05 compared to Ad5+Bud 2 dPi, §p < 0.05 compared to Ad5 2dPi and † p < 0.05 compared to Ad5+Bud 4 dPi.

Apoptosis of viral infected AEC could limit ongoing infection and the resulting inflammation. Infected GPTEC 1 dPi were divided into two pools, one was treated with the corticosteroid Bud, the other not. As demonstrated in Figure 6 viral particle (VP) release into the conditioned media peaked at 2 dPi and then significantly decreased by 4 dPi (2 dPi, 11 × 10^6 ^± 1.8 × 10^6 ^vs. 4 dPi 3.8 × 10 ^6 ^± 1.3 × 10^6 ^§p < 0.05). In contrast, Ad5 infection treated with Bud 1dPi (Ad5+Bud) demonstrated a marked suppression of VP release within 24 hrs of corticosteroid exposure (4.9 × 10^6 ^± 1.2 × 10^6^* p < 0.05). With in the first 24 h of Bud treatment (2 dPi)VP released by the Ad5+Bud group was not different from the untreated pool at 1 dPi. In the subsequent two days VP detection continued to increase in the Ad5+Bud group while the Ad5 alone group demonstrated a significant reduction in VP release § p < 0.05. By 4 dPi there was significantly more viral DNA particles released per day into the media in the Bud treated group when compared to Ad5 alone (8.3 × 10^6 ^± 0.7 × 10^6 ^vs. 3.8 × 10^6 ^± 1.3 × 10^6 ^† p < 0.05). This increase in VP release is coordinate with the altered timing of apoptosis of the Ad5 infected GPTEC in the Ad5+Bud group (Figure 5A). The inset shows amplification of DNA from the conditioned media. Amplification is noted only for Ad5 and not housekeeping genes common to Guinea pigs and human. This result confirmed that the DNA detected by the picogreen assay was indeed Ad5 specific and was not contamination from GPTEC DNA.

### DR4/DR5 expression in the Chronic model of Guinea pig ovalbumin (OVA) induced inflammation and corticosteroid treatment

**Figure 7:**
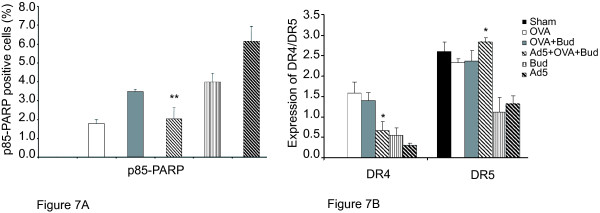
**Guinea pig AEC apoptosis and DR expression as detected by Immunohistochemistry of the Chronic model of Guinea pig viral infection and airway inflammation **Semi-quantitative scoring was utilized to determine the expression of p85-PARP, DR4 and DR5 in the Guinea pig lung sections by immunohistochemistry. Significant reduction in the detection of p85-PARP for Ad5+OVA+Bud group (** p < 0.005) was observed when compared to Ad5 alone group (Figure 7A). However, DR4/DR5 expression for Ad5+OVA+Bud group was higher compared to Ad5 alone group * p < 0.05 (Figure 7B).

Apoptosis and DR4 expression of airway epithelium were associated in the Acute model of viral infection and also demonstrated in the *in vitro *GPTEC model. There was suppressed apoptosis and DR4 and TRAIL expression as a result of Bud treated Ad5 infected cells when compared to Ad5 infection alone. We went on to investigate a model of allergic airway inflammation where persistent viral infection may contribute significantly to the Chronic airway remodeling identified in this condition. The identification of apoptotic cells and death receptor expression was determined as for the Chronic model (Figure 7A, 7B). Apoptosis, as detected by positive staining for the p85 fragment of PARP (Figure 7A) was observed for all the groups except Sham control. Persistent Ad5 infection demonstrated the greater extent of apoptosis (6.1% + 0.78%), followed by Bud (3.9% + 0.48%) and OVA (1.8% + 0.21%) as individual challenges. Bud treatment of OVA- allergic inflammation (OVA+Bud) demonstrated decreased apoptosis compared to Bud only (Figure 7A). Airway epithelial cells positive for p85-PARP were significantly reduced in the Ad5+OVA+Bud group (2.0% + 0.6%) when compared to the Ad5 group (* * p < 0.005)(Figure 7A). A coordinate and consistent response of DR expression to apoptosis was observed only for the groups not infected with Ad5. Sham control had 0% apoptosis and no detectable DR4; OVA and OVA+Bud had increasing apoptosis and DR4 expression. The apoptosis generated by Bud alone is DR independent and thus the reduced DR expression is consistent.

DR4 was not detected at baseline where DR5 was detected at baseline. OVA, Bud, Ad5 individual treatments increases DR4 compared to Sham control, whereas DR5 is unchanged by OVA and decreased by Bud and Ad5 (Figure 7B). OVA+Bud group is unchanged from OVA alone for both DR4 and DR5. DR4 expression is less in Ad5+OVA+Bud compared to OVA+Bud but greater than Ad5 alone. DR5 expression has returned to baseline expression. This demonstrates that each receptor is regulated differently by these challenges.

What is most interesting is the reduced detection of apoptotic AEC in the Ad5+OVA+Bud group. The Ad5 alone has extensive apoptosis with a relatively small but significant increase in DR4 expression relative to baseline and reduced DR5. This suggests the relative importance of DR4 in viral-induced AEC apoptosis. OVA+Bud demonstrate increased DR4 expression and still significant apoptosis relative to Sham baseline. However Ad5+OVA+Bud demonstrates significant increases in both DR4 (0.67 ± 0.22 * p < 0.05) and DR5 (2.83 ± 0.11 * p < 0.05) expression relative to Ad5 alone, but detectable apoptosis is markedly lower than what be expected. As demonstrated in the Acute model, decreased expression of TRAIL the ligand for DR4/ DR5 may account for this effect.

## Discussion

In this study our objective was to determine what role apoptosis and death receptor expression may play in viral infection of AEC. Viral production from infected AEC may be limited by apoptosis, and if dysregulated, in disease states such as asthma, this may lead to longer viral persistence and inflammation. Insight into possible mechanisms for the persistent inflammation would help to develop new therapeutic targets. We studied two models of Acute viral infection and one of asthma post-viral infection of the AEC. This report is the first demonstrating that in the setting of corticosteroid treated inflammation, apoptosis might be dysregulated leading to longer viral persistence. This effect may be mediated by modulation of DR4 and TRAIL regulation in AEC in response to corticosteroid exposure. This resulting apoptosis of AEC suggest a mechanism to limit the viral infection.

Reported differences in DR4 and DR5 expression primarily depend on tissue origin [19-21]. The role for this altered regulation of DR expression in pulmonary tissue and in particular in asthmatics as it relates to epithelial damage, apoptosis, and persistence of viral infection and inflammation is unknown. If DR4 expression is responsible for limiting Acute viral infection by being pro-apoptotic in a model of Acute viral infection of AEC we would expect that DR4 expression and apoptosis would be increased. In Guinea pigs the airway epithelium of uninfected, unsensitized animals does not express immunoreactive DR4 protein (Figure 4B). In contrast, in response to Acute Ad5 infection, DR4 expression in Guinea pig lung tissues increases and is maximal at 3 dPi returning to baseline expression at 7 dPi (Figure 4B). Coordinate with the DR4 expression post-Ad5 infection, apoptosis demonstrated a similar trend as detected by cleaved p85-PARP. The increased apoptosis is compatible with DR4 expression as an initiating factor in Ad5 infection as the detectable DR4 expression precedes apoptosis detection. This is in accordance with the other reports that the death receptor system plays an important role in the elimination of virus-infected cells [7,19,22,23]. Cells infected by human cytomegalovirus, Ad5, reovirus, measles, or HIV demonstrate increased DR4 and DR5 expression rendering them more sensitive to TRAIL-induced apoptosis by autocrine or T-cell derived TRAIL This indicates that DR4 expression corresponds to Acute viral infection leading to apoptosis of infected cells. We confirmed the role for DR4 and TRAIL in an *in vitro *model. Our data demonstrated that in the situation of only Acute Adeno-infection, cultured GPTEC establish an apoptotic pathway via increased receptor (DR4) expression and its ligand (TRAIL) expression, leading to clearance of virally infected cells (Figures 5A, 5B, 5C).

The effects of corticosteroid exposure on Ad5 infection of AEC were investigated further in a cell culture model to determine what role altered AEC apoptosis would have on viral particle production. GPTEC were treated with Bud with or without Ad5 infection. Some DR4 protein expression was observed at baseline for the Sham controls, likely a consequence of epithelial disruption and subsequent culturing but was significantly higher in the Ad5 infected GPTEC (Figure 5B). This is coordinate with the Ad5 induction of DR4 expression in the Acute animal model. Bud alone did not change DR4 expression from baseline but when Bud was added to Ad5 infected cultures DR4 expression was markedly reduced. Bud-induced apoptosis demonstrated the expected steroid effect [24,16] and showed that it is death-receptor independent. However, Ad5+Bud demonstrated suppressed apoptosis and less DR4 expression compared to Bud treatment or Ad5 infection alone. Apoptosis observed as p85-PARP protein expression was less in the early stages of treatment, leading to delayed viral particle production which was compatible with our VP production data in Figure 6. Ad5+Bud group demonstrate "early" vs. "later" effect of induced-apoptosis via suppressed DR4 expression in the setting of corticosteroids. The "shape" of the apoptosis curve as represented by p85-PARP protein in the Ad5 group demonstrate that apoptosis started early is consistent with increased DR4 expression and correlates to early viral particle release which then decreases over time as shown in Figure 6. Ad5+Bud group showed late apoptosis with extended time during which VP release was also detected (Figure 5A, Figure 6). Since the DR4 expression was suppressed in the Ad5+Bud (Figure 5B) group, the maximum p85 protein expression at 4 dPi might be a result of DR-independent Bud effect.

Table 1 data shows that "net" apoptosis by day 4 is approximately the same but timing to onset this apoptosis is altered by Bud. This change in DR4 expression by corticosteroid treatment of viral-infected AEC and the resulting altered regulation of apoptosis would have effects on viral clearance and the resulting inflammation. Initial reduction in VP release in Ad5+Bud group at 2 day compared to Ad5 alone is compatible clinically with corticosteroids treatment used to treat bronchiolitis associated inflammation. This however may be at the expense of altered apoptotic response to viral infection resulting in longer VP production.

The combination of Ad5+OVA+Bud in the animal model generated a condition where apoptosis was decreased (Figure 7A) relative to what was expected considering the response to Ad5 infection in the Acute model (Figure 4A). However, the mechanism established in the Acute animal model (Figure 4A, 4B) and also in the *in vitro *Adeno infection of GPTEC (Figures 5A, 5B) was altered in the case of allergic airway inflammation and corticosteroid exposure where both DR4 and apoptosis are markedly decreased. While there was no change in the DR5 expression in the Guinea pigs of allergic airway inflammation (Figure 7B), DR4 demonstrated a regulation of expression in response to allergic inflammation, viral infection and corticosteroid treatment. DR4 expression was not detected in the control, but was observed in the Ad5 infected/ sham sensitized/challenged group. OVA-sensitized group as allergic model and Bud treatment also demonstrated increase in DR4 expression. This increase may be in response to the OVA-induced airway inflammation, which produces an "asthmatic" phenotype in Guinea pigs characterized by non-specific airway hyper-reactivity and airway eosinophilia [25]. However this increase was not synergistic in combination with Bud and Ad5 treatment. Therefore, DR4 expression and resulting apoptosis may be a mechanism to limit epithelial hyperplasia and metaplasia that can occur in chronic airway inflammation. Uninfected, OVA-sensitized animals have an increase in DR4 and cleaved p85-PARP detection in AEC. *In vivo *DR4 expression as detected in the Ad5 group did not correspond in time to observed apoptotic effect. This effect of time is identified in the *in vitro *GPTEC model too (Figures 5A, 5B). DR expression should correlate to apoptosis and in our model allergic airway inflammation induces not only DR4 expression but the resultant apoptosis of AEC. When the OVA-sensitized animals were treated with the corticosteroid Bud to reduce the eosinophilic inflammation, DR4 expression remained constant relative to that detected in OVA alone. However, the detection of apoptotic AEC by p85-PARP increased dramatically (Figure 7A). The increased apoptosis in the OVA+Bud group reflects the corticosteroid induced apoptosis of AEC as shown previously [24,16] as additive to that generated by inflammation alone. If the mechanism to control AEC survival is dysregulated or disrupted by certain treatments this may contribute to altered cell numbers or other specific structural changes in the airway.

Cells undergo programmed cell death as an initial response to infections by pathogens, such as viruses. The cellular response against viral infection includes production of inflammatory and anti-viral cytokines, as well as the induction of apoptosis. Many viruses have evolved mechanisms that inhibit inflammation and prevent apoptosis and, as a consequence, are able to establish a Chronic phase [26]. DR4 regulation can be the natural mechanism pre-disposing the infected cells to undergo apoptosis. Balance between this pro-apoptotic effect to limit infection in the host and the natural anti-apoptotic viral response will determine the clearance of viral-infected cells and persistence of inflammation. In the animal model, another set of Guinea pigs were Ad5 infected followed by OVA-sensitization and corticosteroid-treatment. This set of experiments demonstrated that DR4 expression is dysregulated by the combination of allergic airway inflammation and corticosteroid treatment in the setting of Ad5 infection. Whereas OVA alone or OVA+Bud increase both apoptosis of AEC (Figure 7A) and DR4 expression (Figure 6B) relative to baseline, the addition of Ad5 suppressed apoptosis and DR4 expression from that detected in OVA+Bud. This reduction in AEC apoptosis could contribute to viral-induced airway inflammation.

Corticosteroids are used clinically to reduce inflammation in viral bronchiolitis and asthma. Reduced viral clearance characteristic in asthmatics [27] may be explained by the corticosteroid use. Recently, decreased apoptosis has been reported in rhinovirus-infected asthmatic airway epithelial cells [28] suggesting an additional innate defect in viral-induced apoptosis of AEC that may also contribute to the asthmatic condition. These are important points as other studies report contradictory results regarding the extent of any anti-inflammatory benefit from corticosteroid therapy. The steroid effects may be dependent on the type of infecting virus [29,30]. Figure 6 demonstrates decreased viral production by infected AEC when treated with corticosteroid only after one day of corticosteroid treatment of the infected culture. Ad5 particle production increased over the subsequent number of days. After 3 days of corticosteroid treatment the GPTEC infected with Ad5 were producing more viral particles than the untreated cultures. The supposed benefit of corticosteroid treatment for viral bronchiolitis may then only be recognized with the first day of treatment and thereafter viral production increases and persists. To what extent this steroid effect contributes to persistent or excessive airway inflammation remains to be determined. These results suggest that DR regulation may be affected by the presence of steroids in the setting of adenoviral infection. To our knowledge, no study has investigated whether viral-induced epithelial damage is improved or worsened by corticosteroid treatment. The overall effects of corticosteroids and how they contribute to the airway remodeling is beyond scope of this study but should be considered in future studies particularly in the setting of viral exacerbations to airway inflammation.

## Conclusion

Our study concludes that in normal AEC, apoptosis is a mechanism to limit viral particle release. DR4 is regulated in response to viral infection. DR4 and TRAIL expression is coordinated with an increase in AEC apoptosis in the Acute model of viral infection and corresponds to virus particle release. However, in the setting of airway inflammation or in the presence of corticosteroids, this mechanism limiting viral particle release is disrupted. Further understanding the mechanism of airway epithelial response to viral infection in the setting of allergic inflammation or steroid exposure would suggest changes in our present therapies for airway inflammation.

## Abbreviations

Death receptors (DR), Adeno virus (Ad5), Guinea Pig Tracheal Epithelial Cells (GPTEC), Airway Epithelial Cells (AEC), Budesonide (Bud)

## Competing interests

The author(s) declare that they have no competing interests.

## Authors' contributions

DRD conceived the idea, DRD and GKS participated in its design, coordination and drafting of the experiments and preparation of the manuscript. KJH contributed by formulation of the initial concept and in editing the manuscript. GKS and TZV participated in the *in vivo *and in vitro sampling, analysis, and statistics, TSC contributed *in vivo *acquisition of data (IHC), analysis and interpretation of data, JYC contributed in cell culturing, western blotting, and PCR studies. TZV and GKS participated in the designing and in vivo sampling for analysis. TSC and JYC were undergraduate students. GKS, TSC, JYC, TZV, KJH, DRD gave final approval for the submission.

## References

[B1] Gern JE (2004). Viral respiratory infection and the link to asthma. Pediatr Infect Dis J.

[B2] Wilson NM (2003). Virus infections, wheeze and asthma. Paediatr Respir Rev.

[B3] Chuang YY, Chiu CH, Wong KS, Huang JG, Huang YC, Chang LY, Lin TY (2003). Severe adenovirus infection in children. J Microbiol Immunol Infect.

[B4] Lyles DS (2000). Cytopathogenesis and inhibition of host gene expression by RNA viruses. Microbiol Mol Biol Rev.

[B5] Kotelkin A, Prikhod'ko EA, Cohen JI, Collins PL, Bukreyev A (2003). Respiratory syncytial virus infection sensitizes cells to apoptosis mediated by tumor necrosis factor-related apoptosis-inducing ligand. J Virol.

[B6] Zhang HG, Xie J, Xu L, Yang P, Xu X, Sun S, Wang Y, Curiel DT, Hsu HC, Mountz JD (2002). Hepatic DR5 induces apoptosis and limits adenovirus gene therapy product expression in the liver. J Virol.

[B7] Clarke P, Meintzer SM, Gibson S, Widmann C, Garrington TP, Johnson GL, Tyler KL (2000). Reovirus-induced apoptosis is mediated by TRAIL. J Virol.

[B8] Barber GN (2001). Host defense, viruses and apoptosis. Cell Death Differ.

[B9] Yamada K, Elliott WM, Brattsand R, Valeur A, Hogg JC, Hayashi S (2002). Molecular mechanisms of decreased steroid responsiveness induced by latent adenoviral infection in allergic lung inflammation. J Allergy Clin Immunol.

[B10] Thomas LH, Sharland M, Friedland JS (2002). Steroids fail to down-regulate respiratory syncytial virus-induced IL-8 secretion in infants. Pediatr Res.

[B11] Azevedo AM, Durigon EL, Okasima V, Queiroz DA, de Moraes-Vasconcelos D, Duarte AJ, Grumach AS (2003). Detection of influenza, parainfluenza, adenovirus and respiratory syncytial virus during asthma attacks in children older than 2 years old. Allergol Immunopathol (Madr).

[B12] Yamada K, Elliott WM, Hayashi S, Brattsand R, Roberts C, Vitalis TZ, Hogg JC (2000). Latent adenoviral infection modifies the steroid response in allergic lung inflammation. J Allergy Clin Immunol.

[B13] Duriez PJ, Shah GM (1997). Cleavage of poly(ADP-ribose) polymerase: a sensitive parameter to study cell death. Biochem Cell Biol.

[B14] Kim JS, Rabe KF, Magnussen H, Green JM, White SR (1995). Migration and proliferation of guinea pig and human airway epithelial cells in response to tachykinins. Am J Physiol.

[B15] Davidson DJ, Kilanowski FM, Randell SH, Sheppard DN, Dorin JR (2000). A primary culture model of differentiated murine tracheal epithelium. Am J Physiol Lung Cell Mol Physiol.

[B16] Dorscheid DR, Wojcik KR, Sun S, Marroquin B, White SR (2001). Apoptosis of airway epithelial cells induced by corticosteroids. Am J Respir Crit Care Med.

[B17] Murakami P, McCaman MT (1999). Quantitation of adenovirus DNA and virus particles with the PicoGreen fluorescent Dye. Anal Biochem.

[B18] Dakhama A, Hegele RG, Laflamme G, Israel-Assayag E, Cormier Y (1999). Common respiratory viruses in lower airways of patients with acute hypersensitivity pneumonitis. Am J Respir Crit Care Med.

[B19] Strater J, Walczak H, Pukrop T, Von Muller L, Hasel C, Kornmann M, Mertens T, Moller P (2002). TRAIL and its receptors in the colonic epithelium: a putative role in the defense of viral infections. Gastroenterology.

[B20] Koornstra JJ, Kleibeuker JH, van Geelen CM, Rijcken FE, Hollema H, de Vries EG, de Jong S (2003). Expression of TRAIL (TNF-related apoptosis-inducing ligand) and its receptors in normal colonic mucosa, adenomas, and carcinomas. J Pathol.

[B21] Spierings DC, de Vries EG, Vellenga E, van den Heuvel FA, Koornstra JJ, Wesseling J, Hollema H, de Jong S (2004). Tissue distribution of the death ligand TRAIL and its receptors. J Histochem Cytochem.

[B22] Lum JJ, Pilon AA, Sanchez-Dardon J, Phenix BN, Kim JE, Mihowich J, Jamison K, Hawley-Foss N, Lynch DH, Badley AD (2001). Induction of cell death in human immunodeficiency virus-infected macrophages and resting memory CD4 T cells by TRAIL/Apo2l. J Virol.

[B23] Servet-Delprat C, Vidalain PO, Bausinger H, Manie S, Le Deist F, Azocar O, Hanau D, Fischer A, Rabourdin-Combe C (2000). Measles virus induces abnormal differentiation of CD40 ligand-activated human dendritic cells. J Immunol.

[B24] Dorscheid DR, Low E, Conforti A, Shifrin S, Sperling AI, White SR (2003). Corticosteroid-induced apoptosis in mouse airway epithelium: effect in normal airways and after allergen-induced airway inflammation. J Allergy Clin Immunol.

[B25] Ishida K, Kelly LJ, Thomson RJ, Beattie LL, Schellenberg RR (1989). Repeated antigen challenge induces airway hyperresponsiveness with tissue eosinophilia in guinea pigs. J Appl Physiol.

[B26] Bensaude E, Turner JL, Wakeley PR, Sweetman DA, Pardieu C, Drew TW, Wileman T, Powell PP (2004). Classical swine fever virus induces proinflammatory cytokines and tissue factor expression and inhibits apoptosis and interferon synthesis during the establishment of long-term infection of porcine vascular endothelial cells. J Gen Virol.

[B27] Papadopoulos NG, Papi A, Psarras S, Johnston SL (2004). Mechanisms of rhinovirus-induced asthma. Paediatr Respir Rev.

[B28] Wark PA, Johnston SL, Bucchieri F, Powell R, Puddicombe S, Laza-Stanca V, Holgate ST, Davies DE (2005). Asthmatic bronchial epithelial cells have a deficient innate immune response to infection with rhinovirus. J Exp Med.

[B29] Grunberg K, Sharon RF, Sont JK, In 't Veen JC, Van Schadewijk WA, De Klerk EP, Dick CR, Van Krieken JH, Sterk PJ (2001). Rhinovirus-induced airway inflammation in asthma: effect of treatment with inhaled corticosteroids before and during experimental infection. Am J Respir Crit Care Med.

[B30] Buckingham SC, Jafri HS, Bush AJ, Carubelli CM, Sheeran P, Hardy RD, Ottolini MG, Ramilo O, DeVincenzo JP (2002). A randomized, double-blind, placebo-controlled trial of dexamethasone in severe respiratory syncytial virus (RSV) infection: effects on RSV quantity and clinical outcome. J Infect Dis.

